# Cancer Stem Cells in Neuroblastoma: Expanding the Therapeutic Frontier

**DOI:** 10.3389/fnmol.2019.00131

**Published:** 2019-05-27

**Authors:** Hisham F. Bahmad, Farah Chamaa, Sahar Assi, Reda M. Chalhoub, Tamara Abou-Antoun, Wassim Abou-Kheir

**Affiliations:** ^1^Department of Anatomy, Cell Biology and Physiological Sciences, Faculty of Medicine, American University of Beirut, Beirut, Lebanon; ^2^Department of Pharmaceutical Sciences, School of Pharmacy, Lebanese American University, Byblos, Lebanon

**Keywords:** neuroblastoma, cancer stem cells, molecular signatures, therapeutic targets, genetic aberrations

## Abstract

Neuroblastoma (NB) is the most common extracranial solid tumor often diagnosed in childhood. Despite intense efforts to develop a successful treatment, current available therapies are still challenged by high rates of resistance, recurrence and progression, most notably in advanced cases and highly malignant tumors. Emerging evidence proposes that this might be due to a subpopulation of cancer stem cells (CSCs) or tumor-initiating cells (TICs) found in the bulk of the tumor. Therefore, the development of more targeted therapy is highly dependent on the identification of the molecular signatures and genetic aberrations characteristic to this subpopulation of cells. This review aims at providing an overview of the key molecular players involved in NB CSCs and focuses on the experimental evidence from NB cell lines, patient-derived xenografts and primary tumors. It also provides some novel approaches of targeting multiple drivers governing the stemness of CSCs to achieve better anti-tumor effects than the currently used therapeutic agents.

## Introduction

Stem cells are a class of multipotent undifferentiated cells, able to give rise to all cells in a particular tissue, organ or organism. Stem cells are selectively characterized by their endogenous self-renewal ability, continuously maintaining a pool of undifferentiated cells that will further differentiate, during development or following injury, into fully functional cells (Reya et al., 2001). Stem cells were historically further classified into two subtypes: pluripotent embryonic stem cells and adult stem cells. The former arises from a fertilized egg and gives rise to whole-organisms, while the latter represents tissue-specific multipotent cells that reside in adult tissues, and maintain their homeostatic balance (Chen et al., 2015). Mainly, these cells regularly divide to replace old senescent cells, or they get rigorously activated following injury to repair damaged tissues (Gudjonsson and Magnusson, 2005; Daley, 2015).

Recently, a new subpopulation of stem cells was suggested to exist in tumors. This hypothesis originated based on the recurrent clinical course of most cancers, as well as their self-renewal ability and uninhibited growth pattern, two categorical markers of tumorigenesis. This subpopulation of cells is commonly referred to as CSC or TICs. The cancer stem cell was first introduced in leukemias, remarkably distinguishing a population of cells on top of the tumorigenesis hierarchy able to develop and generate malignant hematopoietic colonies (Moore et al., 1973). The heterogenic cellular composition of solid tumors, such as melanoma, further supported the existence of CSCs, thought to be responsible for cancer recurrence after therapy (Menaa et al., 2009). As their nomenclature implies, CSCs possess the ability to self-renew indefinitely, as well as the potential to differentiate into the different types of cells that form the tumor bulk. Nonetheless, while normal stem cells are tightly regulated by tumor-suppressor proteins that control their entry into the cell cycle, cellular division and differentiation, CSCs harbor critical genetic pro-oncogenic mutations that instigate uncontrolled cellular proliferation (Chen et al., 2015), promoting tumor progression and malignancy. Notably, in solid tumors such as gliomas, the self-renewal capacity of CSCs (CD133+ cells) correlated highly with the aggressiveness and clinical grade of these tumors (Singh et al., 2003).

Cancer stem cells are also highly resistant to conventional therapies used to treat cancerous tumors, such as chemotherapy and radiation (Reya et al., 2001; Singh et al., 2003). Despite major advances in the delivery, effectiveness and mechanisms involved in these cancer therapies, cancer recurrence and metastatic growth of an eradicated primary tumor are still serious unfortunate outcomes that are commonly seen in clinical settings. Resistance to chemotherapy and treatment relapse are recently being attributed to our failure to successfully target and suppress these CSCs. While common treatments effectively eliminate a large proportion of differentiated cells within the bulk tumor and hence greatly reducing its size and burden to subclinical levels that cannot be detected using conventional methods, it fails to eradicate the tumor-initiating cells, which further promote tumor growth and progression (Menaa et al., 2009).

The field of CSCs has spurred the attention of many researchers and triggered recent studies to focus on further characterizing these cells, on functional and molecular levels. The identification of molecular markers specific to these cells set the stage for the development of novel therapeutic drugs targeted against them, ultimately aiming at preventing tumor recurrence and treatment resistance. The subpopulation of CSCs is found in many types of malignancies including brain tumors (Dawood et al., 2014), which represent some of the most aggressive cancers found in both children and adults.

Neuroblastoma is the most common extracranial pediatric solid tumor. It often arises in infants and children up to 5 years old (Buhagiar and Ayers, 2015). It originates from embryonic neural crest cells destined to become the sympathetic ganglia of the autonomic nervous system, or the catecholamine-secreting cells of the adrenal glands. Thus, these tumors arise mainly in areas such as the adrenal medulla as well as the neck, chest, and spinal cord, among others (Buhagiar and Ayers, 2015). Interestingly, NB tumors with unfavorable prognosis have been shown to house a population of undifferentiated stem cells, responsible of their superior malignant state (Reya et al., 2001; Singh et al., 2003; Buhagiar and Ayers, 2015). In this review, we go through the latest discoveries that outline the molecular markers of the cancer stem cells in NB, as well as the current platform of CSC targeted novel therapies that achieve a higher therapeutic potential than currently used approaches.

## Neuroblastoma Cancer Stem Cells: Molecular Signatures and Genetic Aberrations

Substantial research is aimed at elucidating the molecular signatures of CSCs and specific malignant drivers in order to devise more efficacious therapies. However, the molecular signatures and drivers of malignancy in CSCs seen in solid tumors in general, and NB in particular, are numerous, sometimes overlapping and dynamic. We have previously reported on the RAP in NB that may be rendering it highly malignant and chemotherapy-resistant, a mechanism by which NB CSCs maintain a dynamic ability to revert back-and-forth between anchorage-independent treatment-evasive tumor-spheres and anchorage-dependent, adherent cancer cells in response to environmental stressors, such as therapeutic agents, hypoxia, low pH levels and deprivation of growth factors (Chakrabarti et al., 2012).

Pathways that may be dysregulated by the CSCs have been reported to be involved in invasion and metastasis, uncontrolled proliferation, angiogenic potential, therapeutic resistance and self-renewal abilities (Chakrabarti et al., 2012). Ideal therapeutic strategies necessitate targeting several of these malignant pathways, taking into account the RAP character exhibited by this malignant tumor, in order to adequately cripple the cancer and prevent relapse with metastatic disease. The various, extensively studied molecular signatures pertaining to NB CSCs will be elaborated below (Table 1).

**Table 1:** Summary table of the major molecular signatures and genetic aberrations attributed to neuroblastoma cancer stem cells.

Molecular/Genetic Signature	References	Results
*DLK1*	Kim et al., 2009	*DLK1* expression is induced by hypoxia, via HIF-dependent mechanism, in neuronal tumor cells, and plays a crucial role in maintaining the tumorigenicity of the CSCs.
	Holmquist-Mengelbier et al., 2006	*HIF-1*α and *HIF-2*α enhance *DLK1* expression, but only HIF-2α is differentially expressed in NB with *MYCN*-amplification, whereas *HIF-1*α is ubiquitously expressed.
	Begum et al., 2014	*DLK1* interacts with the cytoplasmic domain of *PHB1* and *PHB2* (via Tyrosine339 and Serine355), the latter of which has a specific and critical role in the self-renewal and clonogenicity of CSCs.
CD114/G-CSFR	Hsu et al., 2013	*CD114+* cells isolated from primary NB tumors are highly tumorigenic and exhibit self-renewal and clonogenic potential, as compared to the *CD114-* population, revealing their stem cell-like phenotype.
	Agarwal et al., 2015	G-CSF acts as an activating growth factor in NB and contributes to the *CD114+* stem cell population through selective activation and upregulation of *STAT3*.
*BMI1*	Cui et al., 2007	Development of primary NB tumors is associated with an increased expression of *BMI1* and its down-regulation is associated with an impaired ability of the NB cells to produce tumors in immunodeficient mice.
*CD44*	Siapati et al., 2011	Inverse correlation exists between *CD44* and *CD24*, which is an established marker of CSCs.
	Jensen et al., 2015; Munchar et al., 2003	*CD44* is a favorable prognostic marker in NB, and lack of *CD44* expression is associated with aggressive and metastatic behavior.
	Rabadan et al., 2013	*CD44* in an inhibitor of metastasis and its downregulation is necessary in NB for the cells to acquire a metastatic potential
*CD133*	Cournoyer et al., 2012	Isolated NB cells with high *CD133* expression show enhanced ability to form neurospheres relative to cells with low expression of *CD133*.
	Takenobu et al., 2011	NB cells lacking *CD133* show loss of ability to repress differentiation and hence were induced to differentiate.
	Sartelet et al., 2012; Tong et al., 2008	*CD133* levels are significantly increased in NB tumors of more advanced stages.
	Tong et al., 2008	NB patients who have increased levels of *CD133* expression show unfavorable histology and shorter survival time post-surgery.
C-kit (*CD117*)	Lau et al., 2015	Increased c-KIT expression is correlated with poor patient prognosis and outcome in NB
	Walton et al., 2004; Ross and Spengler, 2007	I-type NB cells, which highly resemble CSCs, express the *CD117* stem cell marker.
	Ross et al., 2015	Seven genes are identified to be exclusively elevated in NB CSCs, including *CD133*.
*CD24*	Jensen et al., 2015	*CD24* expression is found to be elevated in the metastatic NB.
	Siapati et al., 2011	*CD24* is used as a marker for the NB CSCs.
	Hansford et al., 2007	Primary NB cell lines taken from bone marrow metastases reveal that overexpression of *CD24* glycoprotein in cancer cells is associated with accelerated tumor formation and growth.
		*CD24* is a candidate unique identifier for CSCs in NB, where a small fraction of CD24+ cells are observed within the high-risk NB tumor-spheres derived from bone marrow aspirate
*Fzd6*	Cantilena et al., 2011	*Fzd6* positive cells form more neurospheres compared to the Fzd6 negative cells, indicating their potential CSC phenotype.
*ALDH1*	Flahaut et al., 2016	*ALDH1A2* and *ALDH1A3* are overexpressed in NB CSCs.
		*ALDH1A3* expression is significantly correlated with poor patient prognosis and is associated with tumor progression.
	Coulon et al., 2011	Genes crucial to CSC activity, such as *ALDH1A2*, are upregulated in serial sphere passages derived from patient bone marrow metastatic NB cells.
	Hartomo et al., 2015	High *ALDH1A2* expression is found in patients with poor prognosis.
*LGR5* (GPR49)	Vieira et al., 2017; Shi et al., 2004	*LGR5* is highly expressed in high grade NB tumors.
	Vieira et al., 2017	*LGR5* acts as upstream of MEK/ERK and Akt pro-survival signaling pathways.
*TLX*	Chavali et al., 2014	Under hypoxic conditions, TLX activates both *MMP-2* and *OCT-4* genes, stimulating tumor-sphere self-renewal and promoting migratory abilities of NB cells.
		*TLX* is co-expressed with the migratory neural progenitor markers CD15 and *MMP-2* in xenografts of primary NB cells from patients.
*ABCG2*	Hirschmann-Jax et al., 2004	Primary NB tumor cells have elevated expression of ABC transporters including *ABCG2*, which are responsible for the efflux of therapeutic drugs and subsequent drug resistance and survival of these cancer cells.
Nestin	Garner and Beierle, 2015	Nestin is one of the first markers used in the description of CSCs in NB tumors.
	Thomas et al., 2004	Overexpression of Nestin is linked to aggressive phenotype of NB tumors.
*JARID1B*	Kuo et al., 2015	Depletion of *JARID1B* decreases Notch and its ligand, jagged 1 expression, reduces tumor-sphere formation, inhibits invasion, and enhances chemosensitivity to cisplatin.
*SPDYA*	Lubanska and Porter, 2014	Spy1, a cell cycle regulator encoded by *SPDYA* gene, plays a role in proliferation, self-renewal and differentiation of human NB cells via regulating *CD133*+ cell populations and enhancing neurosphere formation in culture.
		Knockdown of Spy1 causes a decrease in c-MYC expression levels in NB CSCs.
*TRPM7*	Middelbeek et al., 2015	*TRPM7* overexpression reduces actomyosin-driven cytoskeletal tension which promotes SNAI2 expression, a neural crest specifier, and controls the malignant features of NB cells.
	Lange et al., 2017	FTY-720 inhibits *TRPM7* channel activity kinase signaling and at low concentrations, sensitizes drug-resistant NB cells to antineoplastic drugs.
Lamin A/C	Nardella et al., 2015	Down regulation of Lamin A/C in NB cells enhances self-renewal of CSCs *in vitro* and augments ability to initiate tumors *in vivo*.
	Rauschert et al., 2017	Reintroducing Lamin A/C in NB reduces cell growth kinetics and impairs migration, invasion, anchorage-independent cell growth and promotes cytoskeletal restructuring.
		Introduction of lamin Δ50, known as Progerin, drives NB cells into senescence.
*L1-CAM*	Rached et al., 2016	*L1-CAM* is a well-established CSC marker in NB and confers chemo- and radio-resistance in aggressive NB tumors.


### DLK1 and HIF

The *DLK1* gene encodes Protein Delta Homolog 1, a transmembrane protein which belongs to the *EGF*-like homeotic protein family. This gene is highly expressed in different neuroendocrine tumors, including NB (van Limpt et al., 2000; Kim, 2010) and other nervous system tumors such as gliomas (Yin et al., 2006), and plays a critical role in differentiation processes (Laborda, 2000). *DLK1* expression was shown to be induced by hypoxia, via the *HIF*-dependent mechanism (Kim et al., 2009), in neuronal tumor cells and plays a crucial role in maintaining the tumorigenicity of the CSCs (Kim et al., 2009; Begum et al., 2014). *HIF-1*α and *HIF-2*α both enhance *DLK1* expression, however, only *HIF-2*α is differentially expressed in NB with *MYCN* amplification, whereas *HIF-1*α is ubiquitously expressed (Holmquist-Mengelbier et al., 2006; Kim et al., 2009). *DLK1* also interacts with two molecules, PHB1 and PHB2, the latter of which has a specific and critical role in the self-renewal and clonogenicity of CSCs (Begum et al., 2014). In this sense, DLK1 interacts with the PHB complex through its cytoplasmic domain, and Tyrosine339 and Serine355 are specifically required for the maintenance of clonogenicity and tumorigenicity (Kim et al., 2009; Begum et al., 2014).

### CD114/G-CSFR

Granulocyte-Colony Stimulation Factor Receptor expression is well-known as a marker of the CSC population in NB. *CD114+* cells isolated from primary NB tumors were shown to be highly tumorigenic, compared to the *CD114-* population, and exhibited self-renewal and clonogenic potential, revealing their stem cell-like phenotype (Hsu et al., 2013). G-CSF acts as an activating growth factor in NB and contributes to the *CD114+* stem cell population growth through selective activation and upregulation of *STAT3* (Hsu et al., 2013; Agarwal et al., 2015), a pro-oncogenic transcription factor involved in critical cellular functions, such as growth, division and apoptosis. *STAT3* activates *G-CSFR* transcription in a feed-forward manner, contributing to the maintenance and tumorigenicity of the CSC population (Agarwal et al., 2015). Moreover, *G-CSF-STAT3* signaling loop induces the conversion of differentiated cancer cells into CSCs (Hsu et al., 2013). This signaling pathway is also involved in promoting EMT enhancing the migratory and invasive potential of tumor cells (Agarwal et al., 2015).

## BMI1

The oncogene *BMI1* is a transcription factor, member of the polycomb group family that is essential for the self-renewal capacity of stem cells, specifically in the nervous system (Molofsky et al., 2003; Nowak et al., 2006). Development of primary NB tumors is correlated with an increased expression of *BMI1* (Nowak et al., 2006; Cui et al., 2007). On the contrary, *BMI1* down-regulation is associated with an impaired ability of the NB cells to produce tumors in immunodeficient mice (Cui et al., 2007). Moreover, the use of transgenic mice, genetically modified with *MYCN* overexpression, facilitated the monitoring of NB development and revealed an increased expression of *BMI1* (Cui et al., 2007).

Although aggressive NB tumors are associated with *MYCN* amplification, it is important to note that *MYCN* increases the susceptibility of cells to apoptosis (Lutz et al., 1998; Cui et al., 2007) through the *ARF-p53* pathway (Cui et al., 2007). In this context, *BMI1* gene mediates its action by inactivating the apoptotic pathway associated with the *MYN-N* gene amplification, acting as its oncogenic partner (Cui et al., 2007).

### CD44

*CD44* is a transmembrane glycoprotein involved in cell-cell and cell-ECM interactions (Siapati et al., 2011) and was identified as a CSC marker in numerous pediatric tumors, including NB (Mehrazma et al., 2013). Jensen et al. (2015) showed that metastatic NB was characterized with an increased expression of *CD44* marker, compared to non-metastatic controls. Nonetheless, the lack of *CD44* expression was associated with aggressive and metastatic behavior, as well as unfavorable outcomes (Munchar et al., 2003; Siapati et al., 2011; Rabadan et al., 2013; Nardella et al., 2015). *CD44* is involved in cell-cell adhesion, promoting an antagonistic role vis-a-vis cancer metastasis: its downregulation is thought to be necessary in NB for the cells to acquire a metastatic potential (Rabadan et al., 2013). This was further supported with retrospective studies showing a correlation between increased expression of *CD44* and favorable tumors on histology (Munchar et al., 2003). *In vitro* studies on NB cell lines further investigated the role of *CD44* on cellular morphology: whereas *CD44-* cells had smaller size, lesser neuronal projections, and slower growth, *CD44+* cells embraced a more flattened morphology and demonstrated a faster growth, with a protein expression pattern associated with uncontrolled cell cycle progression, immune evasion and lower ability to undergo apoptosis (Siapati et al., 2011). Moreover, one study showed the presence of an inverse correlation between this marker and *CD24* (Siapati et al., 2011), a previously established marker of CSCs (Hansford et al., 2007). The importance of *CD44* as a prognostic marker was challenged by a study conducted by Mehrazma et al. (2013) which failed to prove any correlation between *CD44* expression levels and outcomes in pediatric solid tumors, including NB.

### C-kit (CD117)

*CD117* is an established marker of stem cells (Hirschmann-Jax et al., 2004; Walton et al., 2004) and proto-oncogene (Lau et al., 2015). It is specifically a factor receptor (Hirschmann-Jax et al., 2004) of the tyrosine kinase type (Lau et al., 2015). It was found that increased *C-kit* expression is correlated with poor patient prognosis and outcome in NB (Lau et al., 2015).

The heterogeneous nature of NB tumors has yielded the identification of 3 different phenotypic cell variants when placed in culture (Ross and Spengler, 2007): two phenotypically stable cell variants – the moderately malignant, weakly substrate-adherent N-type neuroblastic cells and the flattened, substrate-adherent non-neuronal S-type cells (Rettig et al., 1987; Ciccarone et al., 1989) – and a third stable cell type that is “intermediate” between N- and S-type cells, termed “I-type” and recognized to be a highly tumorigenic, malignant NB CSC (Ross et al., 1995). Interestingly, only the latter I-type NB cells, which resemble CSCs the most, express the *CD117* stem cell marker (Walton et al., 2004; Ross and Spengler, 2007).

### CD133

*CD133*, also known as prominin-1, is a 120 kD membrane glycoprotein containing five transmembrane domains. While its specific function remains unknown, this molecule is known to be strictly expressed on protrusions of the plasma membrane of epithelial cells among other cell types (Mizrak et al., 2008). It is well studied as a marker of human hematopoietic stem and progenitor cells (Miraglia et al., 1997; Yin et al., 1997), and of neural stem cells (Mizrak et al., 2008). In addition to its expression in normal stem cells, *CD133* is also often used as a marker of CSCs in a variety of tumors, including NB.

*In vitro* studies have linked the expression of *CD133* to tumor-initiating capacities in NB cell lines using the neurosphere formation assay, a functional assay that is used to isolate and assess neural stem cells. *CD133+* cells showed an enhanced ability to form neurospheres compared to *CD133-* cells (Singh et al., 2003; Cournoyer et al., 2012), as well as an increase in average neurosphere size and an enhanced ability of serial passaging, a hallmark of self-renewal (Singh et al., 2003; Cournoyer et al., 2012). Moreover, *CD133+* NB cells generated a significantly higher number of colonies in anchorage-independent conditions (Cournoyer et al., 2012). Furthermore, *CD133+* cells injected into the adrenal gland were able to form tumors while low *CD133-* cells failed to do the same (Cournoyer et al., 2012). Many studies have used *CD133* as a stem cell marker in order to track changes in the proportion of this population of cells upon exposure to certain conditions (Bhaskara et al., 2012; Chavali et al., 2014).

The role of this molecule was further investigated in cancer progression and tumorigenesis. *CD133* knockdown experiments revealed significantly increased differentiation patterns within NB cell lines; *CD133* turned out to play a key role inhibiting differentiation partially through regulating signal transduction downstream of RET tyrosine kinase (Takenobu et al., 2011). Moreover, upon exposure of NB CSCs to signals inducing cellular differentiation, *CD133* expression levels were shown to decrease (Takenobu et al., 2011). On the clinical level, *CD133* levels were seen to be significantly increased in tumors of advanced staging (Tong et al., 2008; Sartelet et al., 2012; Mehrazma et al., 2013). Specifically, patients who were found to have increased levels of *CD133* expression also showed unfavorable histology and a shorter survival time post-surgery (Tong et al., 2008). In support of this, a microarray study was performed on several NB cell lines and revealed the presence of seven different genes whose expression was elevated in the highly tumorigenic “I-type” cells, one of which is *CD133* (Ross et al., 2015). The presence of I-cells in NB tumors is currently recognized as an indicator of tumor malignancy (Walton et al., 2004).

### CD24

*CD24* is a cell adhesion molecule expressed on a variety of cells, such as neural cells and cells of the adrenal medulla, and in several cancers including NB (Akashi et al., 1994). *CD24* expression was found to be elevated in the metastatic NB (Jensen et al., 2015) in one study, and was used as a marker for the NB CSCs in another (Siapati et al., 2011). Additionally, primary NB cell lines taken from bone marrow metastases revealed that overexpression of the *CD24* glycoprotein in cancer cells is associated with accelerated tumor formation and growth (Hansford et al., 2007). Besides, *CD24* had been shown to serve as a candidate unique identifier for CSCs in NB, where a small fraction of *CD24^+^* cells were observed within the high-risk NB tumor-spheres derived from bone marrow aspirates (Hansford et al., 2007).

### Fzd6

A study examining a possible correlation between expression of frizzled receptors and NB prognosis found that only *Fzd6* was directly linked with poor survival in NB patients. Furthermore, *Fzd6* positive cells within these tumors was found to be localized in the tumor hypoxic areas and expressed nuclear *HIF-2*α. The *Fzd6* positive cells were found to form more neurospheres, and were highly invasive and therapy resistant, compared to the *Fzd6* negative cells, indicating their potential CSC phenotype (Cantilena et al., 2011).

### ALDH1 Isoenzymes

Another commonly used marker of stem cells is the subfamily of *ALDH1*, which is composed of *ALDH1A1*, *ALDH1A2*, and *ALDH1A3* isoforms, and are known to be involved in RA synthesis (Koppaka et al., 2012). Specifically, *ALDH1A2* and *ALDH1A3* in NB were found to be overexpressed in a cellular subpopulation of NB, and their roles were further investigated (Flahaut et al., 2016). *ALDH1A2* gene was found to be upregulated using microarray analysis of serial sphere passages derived from patient bone marrow metastatic NB cells, playing a critical role on the self-renewal and stemness of these cells (Coulon et al., 2011). Notably, an increase in *ALDH1A2* expression in NB tumors was found to be correlated with poorer prognosis (Hartomo et al., 2015).

Similarly, *ALDH1A3* expression was also found to be significantly increased in patients with worse outcome, playing a key role in tumor progression (Flahaut et al., 2016). Most importantly, an increase in ALDH activity is directly related to the tumor’s ability to resist conventional drugs and therapies (Abdullah and Chow, 2013; Flahaut et al., 2016).

### LGR5 (GPR49)

The stem cell marker *LGR5*, also known as *GPR49*, is a receptor of R-spondins which a promote canonical Wnt signaling and act as growth factors for stem cells (de Lau et al., 2014). This stem cell marker was found to be overexpressed in several types of cancers, such as glioblastoma (Nakata et al., 2013), cervical (Cao et al., 2017), breast (Yang et al., 2015) and colorectal cancers (Hirsch et al., 2014; Yanai et al., 2017). In addition, *LGR5* was found to be overexpressed in high grade NB (Forgham et al., 2015; Vieira et al., 2017), working upstream of the MEK/ERK and Akt pro-survival signaling pathways (Vieira et al., 2017) which are often triggered in primary NB tumors (Opel et al., 2007).

### MMP

Highly malignant NB tumors acquire the ability to reach blood vessels and metastasize by releasing matrix metalloproteinases, commonly known as MMPs, which are enzymes that allow the cell to degrade its surrounding extracellular matrix (Chavali et al., 2014). In this context, MMP expression was found to be upregulated in advanced-stage NB tumors and more specifically the *MMP-2* and *MMP-9* (or gelatinase enzymes) (Jiang et al., 2011).

### TLX

Nuclear orphan receptor *TLX* (*Drosophila* *tailless* homolog) maintains self-renewal capabilities of neural stem/progenitor cells (Shi et al., 2004) via hypoxia-mediated mechanisms (Chavali et al., 2011). In NB, it has been demonstrated that under hypoxic conditions, *TLX* can activate both *MMP-2* and *OCT-4* genes, stimulating self-renewal of tumor-spheres and promoting migratory abilities of NB cells (Chavali et al., 2014). In this same study by Chavali et al. (2014) *TLX* was co-expressed with the migratory neural progenitor markers *CD15* and *MMP-2* in xenografts of primary NB cells from patients.

### ABCG2

ATP-binding cassette sub-family G member 2 is a marker of neural precursors and is one the first universal markers that were used to identify the CSC population in NB tumors (Ding et al., 2010). *ABCG2* is a member of the *ABC* family of transporter proteins, which are ATP-dependent membrane-spanning proteins involved in the transport of substrates into and out of the cell (Vasiliou et al., 2009). The *ABCG2* transporter was shown to localize in human neural stem/progenitor cells and was proposed to have a crucial role in the maintenance of these cells in an undifferentiated state (Islam et al., 2005).

In a study by Hirschmann-Jax et al. (2004) primary NB tumor cells showed an elevated expression of *ABC* transporters including *ABCG2*, which are responsible for the efflux of therapeutic drugs and therefore for the resistance and survival of these cancer cells. This was further validated by other studies, which showed the role of the *ABCG2* transporter in protecting human neural stem/progenitor cells from toxic substances (Islam et al., 2005).

Even though the specific role of *ABCG2* in cancer progression is not yet known, this protein was shown to be overexpressed in a variety of cancers and numerous reports support its role in the maintenance and tumorigenicity of CSCs. Thus, this marker could have important therapeutic implications in cancer therapies (Ding et al., 2010).

### Nestin

Nestin is an intermediate filament protein that is known to be a marker of neural stem/progenitor cells (Suzuki et al., 2010). It is normally expressed in specific subsets of the mammalian CNS during development, but was also detected in a variety of solid tumors, including NB (Krupkova et al., 2011). A study by Krupkova et al. (2011) demonstrated the localization of the Nestin protein in nuclei of tumor cells. Along with *ABCG2*, it is also one of the first markers used in the description of CSCs in NB tumors (Garner and Beierle, 2015). The overexpression of Nestin was shown to be linked to the aggressive phenotype of NB tumors (Thomas et al., 2004).

### JARID1B

*JARID1B*, also known as *PLU1* or *KDM5B*, is a H3K4me3 histone lysine demethylase identified as an oncogene that is overexpressed in many cancer types (Li et al., 2011; Chicas et al., 2012; Sayegh et al., 2013). A study has associated between *JARID1B* and Notch signaling where depletion of *JARID1B* was found to decrease Notch and its ligand jagged 1 expression, reduce tumor-sphere formation, inhibit invasion, and enhance chemosensitivity to cisplatin (Kuo et al., 2015).

### SPDYA

Spy1, a cell cycle regulator encoded by *SPDYA* gene, plays a role in proliferation, self-renewal and differentiation of human NB cells via regulating *CD133+* cell populations and enhancing neurospheres formation in culture. This atypical cyclin-like protein Spy1, recently shown to be driving symmetric division of glioma stem cells, is a critical factor in the stem-like properties of NB CSC populations (Lubanska and Porter, 2014). On the other hand, knockdown of Spy1 causes a decrease in *c-MYC* expression levels in NB CSCs, a multifunctional transcription factor involved in cellular proliferation (Lubanska and Porter, 2014). In addition, when Spy1 was over-expressed, it hindered the retinoic acid-induced differentiation of NB CSCs. These interesting findings implicate a role for this protein in the regulation of *c-MYC* protein expression that may be driving tumor-sphere self-renewal and maintenance in NB tumors.

### TRPM7

*TRPM7*, a mechanically regulated TRP channel with kinase activity, harbors a crucial role in maintaining progenitor-like gene expression program in human NB cell lines (Clark et al., 2006; Middelbeek et al., 2012). This protein is essential in embryogenesis and the maintenance of undifferentiated neural crest progenitors (Jin et al., 2008, 2012). In NB, *TRPM7* overexpression reduces actomyosin-driven cytoskeletal tension which promotes *SNAI2* expression, a neural crest specifier, and controls the malignant features of NB cells (Middelbeek et al., 2015). More recently a study showed that FTY-720 inhibited *TRPM7* channel kinase activation and signaling and at low concentrations, sensitized drug-resistant NB cells to antineoplastic drugs (Lange et al., 2017). As such, FTY-720 may serve as a chemo-sensitizing agent when used in combination therapy.

### Lamin A/C

Highly proliferative cancer cells have reduced or no expression of Lamin A/C, a major component of the nuclear lamina. This characteristic was verified by enhanced self-renewal of CSCs *in vitro* and increased ability to initiate tumors *in vivo* upon down regulation of Lamin A/C in NB cells (Nardella et al., 2015). More recently, Rauschert et al. (2017) reported that reintroducing Lamin A/C in NB reduced cell growth kinetics and impaired migration, invasion and anchorage-independent cell growth and led to cytoskeletal restructuring. Moreover, the introduction of lamin Δ50, known as Progerin, drove these NB cells into senescence.

### L1-CAM

L1 cell adhesion molecule known as *L1-CAM*, is a well-established CSC marker in various tumors including gliomas (Bao et al., 2008) and NB (Rached et al., 2016) and has been shown to confer chemo- and radio-resistance (Held-Feindt et al., 2012; Rached et al., 2016) in these aggressive cancers. Bao et al. (2008) revealed co-segregation of *CD133^+^* and *L1-CAM^+^* glioma cells where *CD133^+^* glioma cells expressed higher levels of *L1-CAM* compared to *CD133^-^* glioma cells. The *L1-CAM* over-expressing cells also exhibited upregulation of other stem markers including the transcription factor Olig-2. Furthermore, *L1-CAM* over-expression rendered the cancers highly tumorigenic with increased self-renewal and metastatic potential (Bao et al., 2008). Rached et al. (2016) found that *MYCN*-amplified NB cell line IMR-32, representing NB with poor prognosis and lack of response to treatment, revealed significant overexpression of *L1-CAM* compared to non-*MYCN*-amplified SK-N-SH cells. Moreover, the downregulation of *L1-CAM* transcription using siRNA transfection showed significant inhibition of proliferation, migration, and tumor sphere formation, hence suggesting a role in tumorigenicity and maintenance of the CSC sub-population.

## Neuroblastoma Cancer Stem Cells: from Genes to Therapies

### Neuroblastoma CSC Pathways and Proteins That Confer Therapeutic Resistance

Neuroblastoma CSCs have been found to exist in patient primary tumors as well as patient-derived xenograft samples and NB cell lines. This CSC sub-population exhibits high resistance to the currently available therapeutic approaches including chemo-, radio-, and small molecule inhibition therapy (Table 2). These strategies have been successful at eliminating the bulk, non-stem cancer cells, whereas leaving behind the highly aggressive, stem-like TICs.

**Table 2:** Summary table of the major CSC-targeted therapeutic approaches and strategies.

Molecular/Genetic Signature	References	Results
MidKine (MK)	Bilir et al., 2010	Clomipramine and lithium chloride are capable of potentiating vinorelbine cytotoxicity and Midkine, a heparin-binding growth factor, is not a resistance factor for the treatment of neuroblastoma cell lines with the mentioned drugs.
Notch and c-kit	Ayla et al., 2014	Cytotoxic effects of different drugs on neuroblastoma cell lines were not correlated with Notch and c-kit cell signaling.
MDR genes/proteins	Campos-Arroyo et al., 2016	Probenecid co-administered with cisplatin modulate the mRNA and protein expression of the drug efflux transporters MDR1, MRP2 and BCRP
mTOR	Carpentieri et al., 2016	*In vitro* treatment of neuroblastoma cancer cells with mTOR inhibitor (rapamycin) directly differentiates them into osteoblastic and hepatic lineage causing a reversal state of the tumor cells.
Telomerase	Castelo-Branco et al., 2011	Telomerase inhibition exhausts tumor-initiating cells of neural origin.
	Hjelmeland and Rich, 2011	Imetelstat, an oligonucleotide that directly inhibits telomerase activity and is in early clinical development, selectively induces differentiation of neural CSCs and disrupts their growth.
	Wesbuer et al., 2010	Telomerase inhibition increases sensitivity to radiotherapy. Its effect on chemotherapy depends on telomerase activity, the anticancer drug used and the NB cell line.
MAPK	Craig et al., 2016	MAPK inhibition blocks sphere formation in *MYCN*-amplified neuroblastoma cell lines.
Hypoxia	Das et al., 2008	Highly tumorigenic fraction of side population cells migrates to the hypoxic microenvironment in solid tumors *in vivo*.
	Marzi et al., 2007	Treatment of neuroblastoma cell lines with either hypoxia or antiblastic etoposide leads to progressive disappearance of neuronal type cells while maintaining the neural crest stem cells. These cells generate N component cells and fibromuscular progeny. Combination of both modes of treatments cooperated in abolishing the N cells and promoting the conversion to fibromuscular progeny, hence the exhaustion of the tumor.
SLRPs	Farace et al., 2015	Glioblastoma and neuroblastoma CSC-like populations promote increased SLRP activation which induces resistance to temozolomide treatment.
PLK1	Grinshtein et al., 2011	PLK1 inhibitor blocks the growth and survival of neuroblastoma tumor initiating cells in a therapeutic xenograft model.
Proteasome	Hämmerle et al., 2013	Dual therapy of retinoic acid and proteasome inhibitor induced apoptosis, decreased stem cell markers such as Nestin, Sox2 as well as Oct4, and impaired neurosphere formation in neuroblastoma cell lines.
ABDG2 and ABCA3	Hirschmann-Jax et al., 2004	A stable subset of stem cells called “side population (SP)” is identified in primary tumor cells. These SP cells express elevated levels of ABDG2 and ABCA3 and possess increased capacity to expel cytotoxic drugs, thereby developing higher resistance to chemotherapeutic drugs.
G-CSF receptor	Hsu et al., 2013	Isolation of G-CSF receptor-positive subpopulations from primary neuroblastoma tumors or NGP cell line which exhibit high tumorigenicity and capability of both self-renewal as well as differentiation to progeny cells.
Epigenetic modifiers	Ikegaki et al., 2013	Treatment of neuroblastoma cell lines with epigenetic modifiers results in stable malignant stem cell-like NB cells that highly express stem cell markers and have open chromatin structure.
*CD133*	Khalil et al., 2016	The use of the antiepileptic drug valproic acid (VPA) as a histone deacetylase inhibitor with antitumor activities has limitations. Treatment of four human neuroblastoma cell lines with VPA increased *CD133* expression and displayed higher proliferation of cells with lower sensitivity to cytostatic treatment.
	Vangipuram et al., 2010	*CD133*+ NB cells are more resistant to chemotherapeutic drugs than *CD133*- cells.
*DLK1*	Kim et al., 2016	The therapeutic effects of β-carotene on CSCs depend on retinoic acid receptor β which interacts with and downregulates the CSC marker *DLK1*.
	Lee et al., 2013	β-carotene treatment strongly reduces cell growth and induces neuronal differentiation along with downregulation of *DLK1* in neuroblastoma CSCs.
	Lim et al., 2014	In a xenograft model, β-carotene treatment induced tumor cell differentiation and suppressed CSC markers such as Oct 3/4 and *DLK1*. It also down-regulated *HIF-1*α expression and its downstream VEGF.
	Park et al., 2012	Mulberry leaf (ML) extract significantly enhanced the differentiation and reduced sphere formation of neuroblastoma stem cell-like population. Moreover, knock-down of *DLK1* enhanced the inhibitory effect of ML on CSCs.
LIN28/Let-7	Lozier et al., 2015	Difluoromethylornithine (DFMO) treatment on NB cell lines reduced LIN28B and MYCN protein levels, increased Let-7 miRNA and decreased neurosphere formation. DFMO treatment *in vivo* decreased the glycolytic metabolic activity by inhibiting ornithine decarboxylase and restored balance to LIN28/Let-7 axis.
Oct4 and Nanog	Monajemzadeh et al., 2014	Neuroblastoma tumors from 47 patients showed high expression of the stem cell markers Oct4 (23 cases) and Nanog (8 cases), but no strong association between them and the prognostic factors.
BRCA1	Morozova et al., 2010	The profiling of 11 NB TIC lines from 6 NB patients using next-generation RNA sequencing and/or human exon arrays showed frequent mis-expression in the genes of the BRCA1 signaling pathway. The Ingenuity Pathways Analysis tool was applied to predict AURKB drug as a potential novel target for NB.
AMPK	Mouhieddine et al., 2015	Treatment of neuroblastoma cell lines with AMPK pathway activator (Metformin) or inhibitor (Ara-a) significantly reduced the CSCs proliferation and survival in a 2D and 3D tumor-sphere model.
PI3/Akt, RAS-Raf-ERK signaling, p38-MAPK, and TGF-β receptors II and III	Naveen et al., 2016	Neuroblastoma cell lines treated with berberine induced neuronal differentiation, inhibited proliferation, restored tumor suppressor proteins, increased epithelial markers and reverted mesenchymal markers. It instigated reversal of EMT by downregulating PI3/Akt and RAS-Raf-ERK signaling, and upregulating p38-MAPK. It also modulated TGF-β receptors II and III.
EGCG	Nishimura et al., 2012	Epigallocatechin gallate (EGCG), the most abundant catechin in green tea, induced growth arrest and apoptosis in neuroblastoma TICs. It also inhibited sphere formation of parental cells but did not affect the growth of serum-free spheres.
γδ T cells	Nishio et al., 2012	Zoledronate, a mevalonate pathway inhibitor, sensitizes neuroblastoma cell line and the enriched TICs to γδ T-cell- mediated cytolysis. Treatment with both was able to inhibit sphere formation *in vitro* and to decelerate the outgrowth of neuroblastoma TICs *in vivo*.
Intercellular interaction	Choi et al., 2013	Perfluorooctanoic acid (PFOA) and perfluorooctanesulfonic acid (PFOS) decreased the expression of E-cadherin and connexin-43 mRNAs in N2a neuronal cells grown in culture as 2D or 3D.
Endosialin/CD248/TEM1	Rouleau et al., 2011	Endosialin is shown to be expressed in neuroblastoma cell lines, including the CSC-like side population, and in human neuroblastoma xenograft tumors suggesting it to be a suitable therapeutic target.
TrKA	Ruggeri et al., 2014	TrkAIII upregulated SOD2 expression, increased mitochondrial SOD2 activity and attenuated the accumulation of mitochondrial free radical ROS, thereby promoting NB cell line resistance to mitochondrial free radical ROS-mediated death and increasing tumor stem cell-like phenotype.
TAS2Rs	Seo et al., 2017	Increased taste receptors TAS2Rs expression in NB cell lines was associated with increased differentiation, neurite elongation and down regulation of CSC markers with suppressed self-renewal characteristics.
DECA-14 and rapamycin	Smith et al., 2010	DECA-14 and rapamycin induced TIC death *in vitro*, reduced NB xenograft tumor growth and decreased self-renewal and tumor-initiation capacity.
TNKS1	Tian et al., 2014	Inhibition of TNKS1 by small molecule inhibitor or by siRNA knockdown decreased CSC markers and cellular migration ability in *CD133*-isolated neuroblastoma cells.
NDM29	Vella et al., 2015	Over-expression of NDM29 by a small molecule increased the susceptibility of NB cells to cisplatin through decreased ABC transporter expression, responsible for drug resistance.
VEGFRs	Zhang et al., 2009	Sunitinib, a kinase inhibitor of platelet derived growth factor receptors and VEGFRs, inhibited tumor cell proliferation and phosphorylation of VEGFRs in NB cell lines derived from patient tumor samples. In a tumor xenograft model, it inhibited tumor growth, angiogenesis and metastasis. Its use with rapamycin demonstrated synergistic cytotoxicity.
N-myc	Zheng et al., 2013	N-myc-amplified NB cells may become enriched with a CSC-like sub-population after long term drug selection with doxorubicin. Treatment with intermittent low doses of vorinostat downregulates stemness gene expression and sensitizes the drug-resistant cells.
Tubulin polymerization and replicative enzymes	Diaz-Carballo et al., 2014	Different small molecules isolated from Cuban propolis were able to selectively target CSCs from NB tumors in a pleiotropic manner. Of these small molecules, flavonoid was detected, and it disrupts tubulin polymerization, four PPAP-like compounds were isolated and DEHP (CZ6) was determined to inhibit replicative enzymes.


#### Wnt/beta-Catenin Signaling and LRG5

One of the most studied pathways in NB affiliated with therapeutic-resistance is the Wnt/Beta-catenin pathway. Vangipuram et al. (2012) reported an over-expression of genes within the Wnt pathway as well as an increased protein expression of β-Catenin and p-GSK3β in *CD133+* NB cells compared to *CD133^-^* cells. The increased expression of Wnt pathway activity conferred chemoresistance to the cancer stem-like *CD133^+^* cells, while inhibition of Wnt pathway decreased the viability of these cells, ultimately suggesting a protective role for Wnt signaling in the CSC population of NB (Vangipuram et al., 2012). The involvement of Wnt/β-Catenin pathway in inducing therapeutic resistance was further supported by a recent study which showed pro-apoptotic and anti-proliferative effects in an *in vivo* NB model after GSK3 inhibition (Kunnimalaiyaan et al., 2018). This mechanism has also been suggested in other cancers including Wilms’ tumor (Pode-Shakked et al., 2011), colon cancer (Tenbaum et al., 2012), breast cancers (Hallett et al., 2012), pancreatic cancer (Cui et al., 2012) and glioblastoma (Kim et al., 2012).

Leucine-rich repeat-containing G-protein coupled receptors (LRGs) such as LRG5 is important for maintaining Wnt signaling by preventing its ubiquitination and degradation (Hao et al., 2012). Tumor samples from high-risk NB patients not only showed over-expression of Wnt signaling pathway, but also up-regulation of *LRG5* mRNA when grown as tumor-spheres. These tumor-spheres exhibited high expression of Wnt target genes and higher *in vivo* tumorigenicity (Coulon et al., 2011). Moreover, Vieira et al. (2017) demonstrated the important role of *LRG5* in regulating NB survival and proliferation via the MEK/ERK-Wnt/β-Catenin signaling pathways. In fact, MEK/ERK activity has been implicated in the maintenance of embryonic stem cells in the undifferentiated state as well as CSCs in glioma (Kwon et al., 2017), breast cancer (Baker et al., 2018), thyroid cancer (Wang K. et al., 2018), lung cancer (Ning et al., 2018), gastric cancer (Ning et al., 2018), glioblastoma (Wang F. et al., 2018) and rhabdomyosarcoma (Ciccarelli et al., 2016).

Considering the therapeutic implications of these findings, it is tempting to assume that multiple targeting of various tumorigenic pathways may prove beneficial in sensitizing the cancers by eradicating the CSCs within them. In fact, combined targeting of the Wnt signaling with chemotherapy further sensitized the malignant NB cell lines to the treatment, by inducing differentiation. The target genes of the inhibitor, identified by microarray analysis, included *p21*, *p53*, ubiquitin C, *ZBED8*, *MDM2*, *CASP3*, and *FZD1* (Suebsoonthron et al., 2017), thereby explaining the enhanced sensitivity of the malignant NB cell lines to chemotherapy after Wnt signaling inhibition.

#### ABC Transporters

While therapeutic resistance in NB CSCs may be due to several cellular mechanisms, it is likely to be driven by the common MDR mechanism, which is mediated by the ATP-Binding Cassettes (ABC) drug efflux transporters such as MDR1, several MRP and BCRP (Gottesman et al., 2002). Various studies have implicated the role of ABC drug efflux transporters in therapeutic resistance of CSCs.

A recent study reported an increased sensitivity of NB stem cells to combined treatment of the antineoplastic drug (cisplatin) with transporter inhibitor (probenecid) (Campos-Arroyo et al., 2016). The combined treatment led to reduced colony forming capacity of the CSCs, increased apoptosis and decreased proliferation. The authors also reported enhanced caspase-3 activity and significant down-regulation of both mRNA and protein expression of MDR1, MRP2 and BCRP (Campos-Arroyo et al., 2016); therefore, suggesting that the transcriptional inhibition of these proteins prevented drug efflux and allowed the drug to remain longer within the CSCs, ultimately inducing the observed apoptotic effect. Moreover, probenecid decreased the side population of the NB CSCs and reduced the percentage of stem marker CD133 as well as EMT markers vimentin and snail (Campos-Arroyo et al., 2016). In brief, if CSCs are forced back into a “non-stem state” by inducing their differentiation or preventing their EMT progression, therapeutic resistance subsides.

Another study by Vella et al. (2015) demonstrated a reduced growth rate of NB nodules and an increased overall survival of the mice in an animal model treated with chemotherapy (cisplatin) combined with a drug that induces NB differentiation (perhexiline). The pharmacologic induction of NDM29 induced NB differentiation and decreased the protein expression of ABC transporters, specifically in TICs/CSCs thereby increasing the chemotherapeutic sensitivity of this malignant, treatment-evasive NB sub-population.

Other studies have also reported similar findings with NB *CD133+* cells exhibiting up-regulated expression of the ABC transporters and leading to their therapeutic resistance. The possible mechanism by which the *CD133+* cells resist the chemo-therapy may be via up-regulation of ABC transporters that efflux the drug out of the cells (Al-Dimassi et al., 2014), thereby hindering the drugs from inducing their apoptotic effects on the CSCs.

### Signaling Cascades Involved in Tumorigenesis

#### AMPK

The AMP-activated protein kinase pathway has been affiliated with driving the malignant behaviors of many tumors including brain cancers (Rehman et al., 2014; Li et al., 2017). Drugs that target this pathway have been investigated to elucidate their role in eliminating cancerous growth. Stem-like TICs within the bulk of tumors have been found to respond to such therapies. A study by Mouhieddine et al. (2015) demonstrated that Metformin, an AMPK activator and Ara-a, an AMPK pathway inhibitor both significantly reduced NB and glioma CSC proliferation and survival in a 2D and 3D tumor-sphere model. The importance of this finding lies in the fact that while the two drugs work antagonistically to each other, their anticancer effects may be due to the inhibitory effects on the mTOR pathway and Akt achieved with Metformin, and the energy deprivation achieved with Ara-a-induced inhibition of AMPK, thereby starving the CSCs. Furthermore, being a nucleoside analog, Ara-a may induce anti-cancer effects by interfering with DNA synthesis in these cells. In fact, the authors report the higher potency of Ara-a on these cancers (Mouhieddine et al., 2015).

#### MAPK and PI3K/mTOR

Various studies have demonstrated the efficacy of mTOR pathway inhibition on reducing tumor growth and specifically inhibiting proliferation of CSCs. Smith et al. (2010) reported that rapamycin, an mTOR inhibitor, successfully decreased cell viability and proliferation in an *in vitro*, NB patient-derived CSCs model. This activity was mediated by dephosphorylation of mTOR downstream targets p70^S6K^ and S6RP. We further validated this finding in a recent study from our lab, where triciribine and rapamycin were used to study the role of inhibiting two different points of the Akt/mTOR pathway *in vitro* on U251 (glioblastoma) and SH-SY5Y (NB) human cell lines and their CSCs (Bahmad et al., 2018). Both drugs decreased the SFU of glioblastoma and NB cells thus extinguishing their CSC populations (Bahmad et al., 2018). On the other hand, apoptotic markers, such as cleaved PARP and increased sub-2n DNA were observed after rapamycin treatment in NB CSCs (Smith et al., 2010). In addition, it has been reported that NB CSCs infected with mTOR shRNA demonstrated 70–80% growth inhibition compared to mock-infected NB CSCs. Daily *in vivo* rapamycin treatment of mice bearing NB tumors successfully reduced tumor weight by >70% compared to only 43.4% achieved with vinblastine treatment, the standard chemotherapeutic drug used for NB. Tumor-sphere formation in the NB CSCs was also reported to be significantly (7.75-fold) inhibited after rapamycin treatment compared to vehicle-treated CSCs, thereby implicating the CSC-targeting ability of rapamycin (Smith et al., 2010).

As previously well established, *P53* functions to suppress mTOR pathway under stressful conditions in cells. When cells undergo stress, the functional P53 protein triggers transcription of proteins that negatively regulate the IGF/AKT/mTOR pathway to induce cell-cycle arrest, DNA repair, senescence or apoptosis, depending on the stressor and tumor type (Moreno-Smith et al., 2017). As such, combination therapy that stabilizes P53 expression along with mTOR inhibitors may prove more efficacious than single therapy that often leads to resistance. Moreno-Smith et al. (2017) reported on the enhanced effects of temsirolimus (mTOR inhibitor) when combined with *P53* activators (Nutlin 3a, a first-generation and RG7388 a second-generation MDM2 inhibitor) in an *in vivo* model of NB. The combined therapy enhanced the anti-proliferative and pro-apoptotic effects of single-agent therapy, implicating the importance of *P53* stabilization in inducing mTOR inhibition and subsequent cellular apoptosis. This was further tested in an orthotopic, *in vivo* pre-clinical model using both *MYCN*-amplified and non-amplified cell lines. In both cell lines used, the combination therapy was significantly more potent in anti-tumor activity compared to either monotherapy. More importantly, the combination therapy led to long-term cessation of tumor growth after treatment withdrawal by induced apoptosis (Moreno-Smith et al., 2017).

### Hypoxia and Retinoic Acid

The common link between mTOR, PI3K and insulin-like growth factor (IGF) in NB is hypoxia (Påhlman and Mohlin, 2018). In fact, hypoxic regions of NB have been reported to express the hypoxia-inducible factors 1 and 2 (*HIF1* and *HIF2*), which coincidently, confer stem-like features in these cells, including immature, neural-crest like cells with self-renewal potential. In addition, *HIF2* and IGF co-expression is correlated in clinical NB specimens, and *IGF* regulates hypoxic expression of *HIF2* (Påhlman and Mohlin, 2018). Moreover, IGF receptor binding induces PI3K signaling which subsequently drives tumorigenesis such as cancerous growth, survival and differentiation. PI3K and mTOR activation by growth factors also initiates *HIF1* translation, therefore the PI3K/mTOR pathways are putative candidate mediators of IGF-driven *HIF* expression and activity in NB and CSC maintenance and proliferation (Påhlman and Mohlin, 2018).

Inducing differentiation of NB CSCs may render them more sensitive to therapeutic intervention. Retinoic acid is clinically used to induce cancer differentiation in patients that have undergone induction and consolidation chemotherapy for high-risk NB (Duffy et al., 2017). RA (13-*cis*-retinoic acid) is a well-known differentiating agent reported to reduce stemness characteristics in various tumors including NB (Craig et al., 2016). Cimmino et al. (2015) demonstrated that *HIF1A* silencing combined with ATRA treatment led to differentiation and senescence of NB cells into a more benign, glial lineage that would render them therapeutically responsive.

Interestingly, combined RA treatment with proteasome inhibition using MG132 led to growth arrest and differentiation, inhibition of tumor-sphere formation and apoptosis in NB CSCs. Stem markers Nestin, Sox2, and Oct-4 were all reduced with the combination therapy as compared to monotherapy (Hämmerle et al., 2013), exemplifying the importance of differentiation inducers used together with other agents to enhance the anti-CSC effect in the malignant, sub-population of these NB tumors.

### Epigenetics

The vast majority of NB tumors in children arise due to somatic mutations, as opposed to germline mutations of the anaplastic lymphoma kinase (ALK) gene in familial neuroblastoma, accounting for 2% of the cases (Mosse et al., 2008). Nonetheless, epigenetic regulation has been the suspected culprit believed to play a critical role driving these malignancies. The heterogeneity of NB tumors is therefore believed to be driven by a combination of somatic mutations and epigenetically-regulated factors. In fact, epigenetic modifications have been reported to induce a stem-like cancer cell in NB (Muñoz et al., 2012; Kobayashi et al., 2013; Toh et al., 2017). Specifically, the epigenetically-induced NB CSCs exhibited high expression of stemness factors and stem cell markers, they had open chromatin structure, increased tumor-initiating ability and metastatic potential. Moreover, they had a highly undifferentiated histological appearance with over-expression of *MYC/MYCN* (Ikegaki et al., 2013).

*MYCN* has been shown to interact with epigenetic machinery (He et al., 2013) and Duffy et al. (2017) reported that epigenetic regulators, including HDACs and BRD4, were differentially activated in *MYCN*-amplified NB, affecting the RA-induced differentiation in these cells. The authors reported that RA inhibits *HDAC* functioning while *MYCN* overexpression activates it. An important regulator identified in this study was *TGFB1*, a ligand for TGF-β signaling pathway. Pharmacological activation of the TGF-β signaling pathway using KGN, a small molecule that indirectly enhances TGF-β signaling by regulating the activity of SMAD transcriptional effectors, was used in combination with RA to determine the effect of this dual therapy on NB differentiation and malignancy. Both compounds used as sole therapy yielded a modest differentiation response, but in combination, exerted a more potent response and reduced viability in the *MYCN*-amplified NB cells (Duffy et al., 2017), thereby signifying the importance of using combination therapy in *MYCN*-amplified, high-risk NB patients to reach better clinical outcomes.

Others have also shown that *HDAC* inhibition via vorinostat in *MYCN*-amplified NB cells reduced chemo-resistance (DOX response) and *in vitro* invasive potential, inhibited tumor-sphere formation and down-regulated stem marker expression in NB CSCs (Zheng et al., 2013). And yet another group reported on the synergistic effect of combining RA with the *HDAC* inhibitor CBHA in an *in vivo*, human xenograft NB model (Coffey et al., 2001). This was achieved via the inhibition of AKT signaling pathway and survivin (Shah et al., 2013), whose expression usually promote tumor cell proliferation and inhibit apoptosis.

Moreover, combined therapy with *HDAC* inhibitors and ATRA down-regulated c*-Myc*, the neuronal markers *NeuN* and β-3 tubulin, as well as the oncoprotein *BMI1*, and more potently reduced cellular proliferation in NB cell lines (Almeida et al., 2017). Knowing that *BMI1* is a potent stem cell maintenance oncoprotein, this combination therapy may prove effective due to eliminating the CSC sub-population in the aggressive form of NB. As previously reported, *BMI1* is capable of repressing *P53* responses in NB precursors leading to NB initiation (Calao et al., 2012). Furthermore, *BMI1* can directly bind to p53 in a complex with other Polycomb complex proteins, Ring1A or Ring1B, leading to increased p53 ubiquitination and degradation. Another study revealed that *BMI1* expression positively correlated with a nucleotide binding protein BORIS/CTCFL, found to be aberrantly expressed in various malignancies and primarily re-expressed in CSCs (Garikapati et al., 2017). The authors of this study demonstrated an important cross-talk between BORIS/CTCFL and Wnt/β-catenin signaling pathways that led to enhanced expression of stemness proteins and increased EMT markers such as N-CAD, Vimentin and SNAIL. This intricate network of communication between *BMI1*, *BORIS/CTCFL* and *Wnt* signaling plays a crucial role that confers therapeutic resistance and malignancy in the aggressive *MYCN*-amplified NB cell line.

### Delta-Like 1

Another important player in hypoxia-induced cancer stemness, as mentioned above, is the *DLK1*, which has been shown to be up-regulated after hypoxia induction in neuronal tumors (Kim et al., 2009). Its protein up-regulation enhances tumor stemness and tumorigenic growth *in vivo*, whereas its silencing drives cellular differentiation and reduces the tumorigenic potential in NB CSCs (Begum et al., 2014).

The combined treatment of NB cells with RA and *DLK1* knock-down led to significantly higher levels of cellular differentiation compared to monotherapy with either agent. Interestingly, the *DLK1* effects seem to be mediated via prohibition *PHB1* and *PHB2* interactions which have previously been reported to play roles in cellular proliferation, apoptosis, transcription, mitochondrial folding and cell signaling (Theiss and Sitaraman, 2011). The authors in Begum et al. (2014) demonstrated that the interaction between *DLK1* and both *PHB1* and *PHB2* via its cytoplasmic domain, bring about NB cell stemness and tumor-sphere self-renewal. They showed that knock-down of either *PHB* or *DLK1* reduces the clonogenic and self-renewal potential of NB CSCs. This interaction was a novel finding and thus, an important avenue to explore in multi-targeted approaches aimed at eliminating the CSCs within the bulk of the tumor along with the differentiated, non-stem cancer cells.

### G-CSF/STAT3

Granulocyte-colony stimulation factor receptor positive or *CD114+* NB cells have stem-like characteristics that render them highly tumorigenic, self-renewing, with a treatment evasive phenotype (Hsu et al., 2013). Acting via *STAT3* stimulation and up-regulation, G-CSF presents itself as a very attractive target for NB CSC elimination and for increasing the potential long-term cancer-free survival in children. In fact, Agarwal et al. (2015) demonstrated that anti-G-CSF antibody or *STAT3* inhibition led to depletion of CSC subpopulation within tumors which was correlated with tumor growth inhibition, decreased metastasis, and increased chemo-sensitivity in a pre-clinical NB animal model. Such an observation warrants the consideration of a multi-modality therapeutic approach that targets the G-CSF-STAT3 signaling in the NB CSCs together with the standard chemotherapeutic drugs in order to completely eradicate the high-risk NB.

Gheeya et al. (2009) had previously demonstrated that *STAT3* inhibition using Cucurbitacin I induced an antitumor effect in various solid tumors including NB with and without *MYCN*-amplification. More recently, Honokiol, a biphenolic natural product isolated from the bark and leaves of Magnolia plant, and an inhibitor of the *STAT3* signaling pathway was reported to inhibit cancerous growth in various tumors including NB (Prasad and Katiyar, 2016). Yet another natural inhibitor of *STAT3* called curcumin or turmeric (diferuloylmethane), also known as an inhibitor of *Akt*, *COX2* and *NF-κB* signaling, demonstrated anti-cancer properties which rendered it a chemo-sensitizing agent against various tumors including glioma, NB, cervical carcinoma, epidermal carcinoma, prostate cancer, and colon cancer (Goel and Aggarwal, 2010).

### L1-CAM

One of the prominent players in driving tumorigenic invasion and motility is *L1-CAM* (Zaatiti et al., 2018) whose suppression was explored as a therapeutic strategy in various tumors. Bao et al. (2008) reported that shRNA knock-down of *L1-CAM* in *CD133^+^* glioma cells significantly reduced neurosphere self-renewal potential, increased apoptosis, but inhibited proliferation of CSCs. *L1-CAM* silencing suppressed tumor growth and increased survival in an *in vivo* xenograft mouse model (Bao et al., 2008). This silencing was associated with reduced expression of *Olig2*, but increased expression of the *p21* (*WAF1/CIP1*) tumor suppressor, suggesting their possible involvement in the *CD133^+^* driven mechanistic effects in glioma cells.

The CE7 epitope of L1-CAM (CD171) has been explored in the context of CAR-T therapy in NB tumors (Hong et al., 2014; Kunkele et al., 2017) and other cancers, including lung, ovarian (Hong et al., 2016), and renal carcinomas as well as glioblastomas (Hong et al., 2014). One study concluded that CE7 epitope of L1-CAM may be amenable to CAR-T targeted therapy and thus serves as an invaluable tool in adoptive immunotherapy (Hong et al., 2014). Moreover, in a more recent pre-clinical assessment, (Kunkele et al. (2017) tested the safety of targeting the CE7 epitope CD171 with CE7-CAR T cells and determined whether bioactive CAR-T cells may be generated from heavily pretreated NB patients with recurrent or refractory disease. Fortunately, the authors confirmed the efficacy and safety of the CE7 epitope on CD171 to be used as a CAR-T cell target for NB patients with recurrent/refractory disease (Kunkele et al., 2017).

### Polo-Like Kinase 1 (PLK1)

Neuroblastoma CSCs have been previously reported to highly express the serine/threonine kinase, Polo-like kinase 1, a known inducer of G2/M-phase transition. Grinshtein et al. (2011) demonstrated that the PLK1 inhibitor, BI 2536 significantly reduced tumor growth in an *in vivo* xenograft therapeutic model of NB CSCs when administered alone or in combination with irinotecan, which is typically used in patients with refractory NB.

More recently, Pajtler et al. (2017) investigated the tumorigenic effects of a PLK1 competitive inhibitor, GSK461364, in both *in vitro* and *in vivo* NB models. This treatment led to significant reduction in cell viability and proliferation and it also induced cell cycle arrest and apoptosis. In addition, the growth of established xenograft tumors in nude mice was significantly delayed, whereas survival time in the treatment group significantly increased (Pajtler et al., 2017). This further highlights the importance of targeting the CSC drivers in an attempt to achieve better anti-tumor effects of the currently used therapeutic agents.

## Conclusion and Future Directions

A pressing challenge of nervous system tumors in general and NB, specifically, is to overcome the therapy-resistant nature of CSCs. The eminent role they play in aggravating chemoresistance and malignancy magnifies the need to identify (1) the molecular signatures and the genetic aberrations of CSCs in neuroblastoma and (2) the therapeutic intervention that has been used in targeting the stem cell dysregulations. Even though a unique set of CSC markers have not been identified yet, a group of molecules have been associated with a stem-like behavior in cells expressing them, on the molecular, cellular and functional levels. It is foreseeable that targeting the key players that confer stemness to CSCs can substantially eliminate the CSC-like phenotypes. Moreover, combination of multiple targeting strategies provides a potential therapeutic anti-cancer intervention that needs to be further tested and studied. In the field of neuroblastoma, the ongoing efforts in eradicating CSCs may allow for promoting the survival of the children in ways comparable to the progress seen with some leukemias (He et al., 2015; Al-Hussaini et al., 2016; Ruella et al., 2016; Desai et al., 2019).

## Author Contributions

All authors worked on study conception and design, analyzed and interpreted the data, drafted the manuscript, and read and approved the final draft. HFB and WA-K screened titles for relevance and abstracted the data from the eligible full text articles. TA-A and WA-K critically revised the manuscript with input from the entire team.

## Conflict of Interest Statement

The authors declare that the research was conducted in the absence of any commercial or financial relationships that could be construed as a potential conflict of interest.

## Abbreviations

ABC: adenosine triphosphate-binding cassette
ABCG2: ATP-binding cassette sub-family G member 2
ALDH1: aldehyde dehydrogenases 1
ATRA: all-trans retinoic acid
BCRP: breast cancer resistance protein
CBHA: carboxycinnamic acid bis-hydroxamide
CSC: cancer stem cell
DLK1: Delta-like 1
EMT: epithelial-to-mesenchymal transition
Fzd6: frizzled receptors 6
G-CSFR: Granulocyte-Colony Stimulation Factor Receptor
HIF: hypoxia-inducible factor
KGN: kartogenin
MDR: multidrug resistance
MDR1: P-glycoprotein
MMP: matrix metalloproteinase
MRP: multidrug resistance proteins
NB: neuroblastoma
NDM29: neuroblastoma differentiation marker 29
PHB1: prohibitin 1
PHB2: prohibitin 2
PLK1: Polo-Like Kinase 1
RA: retinoic acid
RAP: reversible adaptive plasticity
S6RP: S6 ribosomal protein
SFU: sphere-forming units
STAT3: Signal Transducer and Activator of Transcription 3
TIC: tumor-initiating cell
